# Multi-level combinatoriality in magpie non-song vocalizations

**DOI:** 10.1098/rsif.2022.0679

**Published:** 2023-02-01

**Authors:** Sarah L. Walsh, Sabrina Engesser, Simon W. Townsend, Amanda R. Ridley

**Affiliations:** ^1^ Centre for Evolutionary Biology, School of Biological Sciences, University of Western Australia, Crawley, WA 6009, Australia; ^2^ Department of Biology, University of Copenhagen, 1165 København, Denmark; ^3^ Department of Comparative Language Science, University of Zurich, Zurich 8006, Switzerland; ^4^ Center for the Interdisciplinary Study of Language Evolution (ISLE), University of Zurich, Zurich 8006, Switzerland; ^5^ Department of Psychology, University of Warwick, Coventry CV4 7AL, UK

**Keywords:** animal communication, multi-level combinatoriality, call combinations, UMAP, Western Australian magpie

## Abstract

Comparative studies conducted over the past few decades have provided important insights into the capacity for animals to combine vocal segments at either one of two levels: within- or between-calls. There remains, however, a distinct gap in knowledge as to whether animal combinatoriality can extend beyond one level. Investigating this requires a comprehensive analysis of the combinatorial features characterizing a species' vocal system. Here, we used a nonlinear dimensionality reduction analysis and sequential transition analysis to quantitatively describe the non-song combinatorial repertoire of the Western Australian magpie (*Gymnorhina tibicen dorsalis*). We found that (i) magpies recombine four distinct acoustic segments to create a larger number of calls, and (ii) the resultant calls are further combined into larger call combinations. Our work demonstrates two levels in the combining of magpie vocal units. These results are incongruous with the notion that a capacity for multi-level combinatoriality is unique to human language, wherein the combining of meaningless sounds and meaningful words interactively occurs across different combinatorial levels. Our study thus provides novel insights into the combinatorial capacities of a non-human species, adding to the growing evidence of analogues of language-specific traits present in the animal kingdom.

## 1. Introduction

The level of complexity present in human language appears unparalleled in the animal kingdom [1,2] and has led to contentious debate surrounding its evolutionary origin [3–6]. In efforts to resolve this uncertainty, the past half-century has seen a rise in comparative studies investigating the extent to which language-specific traits are also present in more simple forms in non-human animals [7–9]. Such research has revealed analogues of traits, previously thought to be uniquely human, are also observed in several lineages (including *functional referentiality* [9–11], *intentionality* [12,13] and *vocal learning* [14,15]). These studies highlight the importance of the comparative research method when investigating language's origin, as a valuable tool to provide insight into the evolutionary paths of language-specific abilities [16–18]. However, the cryptic evolutionary history of one fundamental language trait remains less clear: the capacity for open-ended combinatorial generativity [4,6,19].

Language's open-ended generativity is a product of combinatorics on two distinct levels: combining non-meaningful sounds into meaning-bearing vocal units (words; *phonology*), and the combining of words into phrases, wherein phrase meaning is derived from the meanings and order of its comprising words (*syntax/compositionality*) [20]. Historically, a lack of comparative data led to a long-standing notion that the capacity for unbounded combinatoriality is the defining feature separating humans from other animals [2]. Additionally, foundational comparative research only furthered this notion through (i) initial unsuccessful searches for examples in species closely related to humans, or (ii) focusing on animal song, which arguably bears little resemblance to language's level of combinatorial complexity due to the lack of context-specific meaning [21–24]. In recent years, a shift in focus to include distantly related lineages and a broadening toward studies on non-song vocalizations has revealed that the capacity for combinatoriality may be widespread in the animal kingdom [5,7,25,26]. Chestnut-crowned babblers (*Pomatostomus ruficeps*), for example, combine two meaning-devoid sounds (A and B) into a pair of functionally distinct calls: AB-flight calls, which relate to group movement, and BAB-prompt calls, which stimulate nestling begging [27,28]. Given that the calls are composed of meaningless elements, and that the modification of element arrangement denotes meaning differentiation, this example bears at least some resemblance to simple phonology in human language [28].

On the next combinatorial level, meaning-derived call combinations have been described in two distantly related bird species: Japanese tits (*Parus minor*) and pied babblers (*Turdoides bicolor*). In both species, alert and recruitment calls are combined when mobbing predators [29,30]. Importantly, the meaning of the combination can be derived from the meanings of its comprising calls, thus representing a rudimentary form of compositional syntax analogous to that observed in human language [5,31,32].

There is evidence for various other forms of combinatoriality in species (within and outside the avian lineage), many of which can be considered analogies of language components (for a detailed review see [7]). Collectively, these studies suggest that mechanisms for combinatoriality, albeit rudimentary, exist across a diverse range of taxa [5,32,33]. These studies, however, fall short of providing evidence that bears resemblance to the extent of combinatorial complexity observed in language. Critically, no study to date has provided compelling evidence for any non-human species possessing a capacity to vocally combine meaningful information on more than one level, as seen in language [4,7,32,34]; but see [35]. Quantitative analyses, however, rarely incorporate the full repertoire for a species and, thus, leave potential for the extent of combinatorial capacity to remain hidden.

Our study aims to investigate the potential for complex combinatoriality in a highly vocal bird: the Western Australian magpie (hereafter magpie). Magpies are found in southwestern Australia and live in year-round groups within relatively stable territories [36,37]. The vocal repertoire of mature magpies includes a wide range of call types, such as alarm calls, distress or alert calls, feeding grunts, song and mimicry [38–42]. Natural observations suggest that discrete calls and combinations possess context specificity and evoke stereotyped responses (including calls associated with alert and recruit-type events [42]).

Previous work has shown a capacity for combinatoriality in magpies, with results indicating that two independently produced calls are commonly combined into a higher level vocalization [40]. Building on this work, pilot investigations for the current study suggested that magpies may possess additional complexity in the structuring of call combinations, and that the comprising calls may further be composed of smaller vocal units. Investigating the sequential relationships between vocal units making up calls and call combinations may help to shed light on the origins of complex combinatoriality and the selective pressures driving this evolution across taxa [7,26]. Additionally, although this paper does not focus on the function or meaning of magpie vocal units, we suggest knowledge may be gained here through investigating whether vocal production and arrangement varies across different groups and individuals. The reason being, if there is little individual or group difference in the acoustic properties within vocal unit classes, and sequential transition between classes is similarly carried out across groups and individuals, there is a suggestion for these components of the communication system to hold functional relevance to the species. Consequently, we suggest this investigation can also reveal the likelihood of whether vocal units and combinations encode meaning that is relevant to the receiver [7,26].

In this study, we use an unsupervised nonlinear dimensionality technique (uniform manifold approximation and projection, hereafter UMAP) to obtain a comprehensive understanding of the structure and variation of vocal units comprising the magpie combinatorial repertoire. Additionally, we investigate sequential relationships between vocal units to gain insight into the predictability of vocal unit arrangement in calls and call combinations. By that means, we can determine whether vocal production and sequential transition is consistent across individuals and groups, which, in turn, can provide insight into the functional importance of combinatoriality in this system.

## 2. Methods

### 2.1. Study population

Data were collected from 18 magpie groups located in the Perth urban suburbs of Crawley (31.9752° S, 115.8213° E; *N*: 7 groups) and Guildford (31.8994° S, 115.9717° E; *N*: 11 groups) located roughly 15.4 km apart. Groups ranged in size from 2 to a maximum of 14 adult (greater than 2 years old) individuals and have been observed for several years (ranging from 3 to 9 years). Individuals are thus habituated to observer presence, allowing data collection from a close distance (approx. 5 m). Individuals have been ringed for identification or are otherwise identified through distinctive morphological features (e.g. scarring and plumage anomalies). All field methods were approved by the University of Western Australia Ethics Committee (Protocol number: RA/3/100/1656 and 2021/ET000272).

### 2.2. Data collection and selection

Recordings were conducted between October 2014 and September 2021. Vocalizations were recorded at a 44.1 kHz sampling rate using either a Roland R-05 or Roland R-07 digital WAV recorder linked to a RODE NTG2 directional condenser microphone encased in a RODE blimp suspension windshield. Data were primarily collected during 4 to 10 h sessions when this species is most active (between sunrise and 14.00; [43]) and randomized between groups and individuals within this timeframe. Most recordings were taken during focal animal sampling, where a focal individual was followed for a period ranging from 20 min to 1 h, at a distance of 5–10 m [44,45]. If birds displayed any sign of heightened levels of behaviour following arrival at the group, onset of focal sampling was delayed until the group returned to normal behaviour (usually 5 min). Additional vocalizations produced by individuals within close range (less than 10 m) of the microphone (regardless of whether this occurred during focal animal sampling) were recorded ad libitum to increase sample size.

The total sound database comprised 3201 recordings, cut from raw files (including focal recordings) tallying over 370 h. Data considered for this study were limited to recordings containing non-song call combinations produced by a known signaller. Only non-song call combinations were included due to the aim of obtaining a statistical measure for the acoustic structural variation in vocal units comprising combinatorial structures. Data were further restricted to all recordings taken from up to one adult male and female per group, based on those individuals with the highest count of recordings. Thereby, we allowed for consideration of any sex differences and also ensured that no one group was over-represented, which could bias the results.

Spectrograms were assessed visually using the sound editing software Audacity(R) v. 3.0.2 [46]. Most audio files selected for analysis contained high-quality recordings of combinations (based on high signal-to-noise ratio) that were undisturbed by other sounds. However, to further increase quality in signals slightly corrupted by background noise, we used the noise reduction function in Audacity(R) which applies a filter to the signal based on a noise profile taken from the same recording (3 s noise sample, set by the user). Noise profile filtering has been found to be effective at reducing noise in avian vocal signals, while largely conserving the quantitative features of the signal, in cases where noise is constant over the signal duration and signal intensity exceeds that of noise [47]. Call combinations were located by listening to the recording and inspecting the corresponding spectrogram. We obtained a larger number of recordings for females compared with males, which is consistent with previously recorded female-biased sex differences in magpie singing behaviour [38]. We did not set a minimum threshold on the number of recordings or combinations per individual. The final database used in analysis comprised 153 recordings which contained 273 combinations by 15 females and 8 males from 16 groups [48].

### 2.3. Sound segmentation

**Figure 1.**
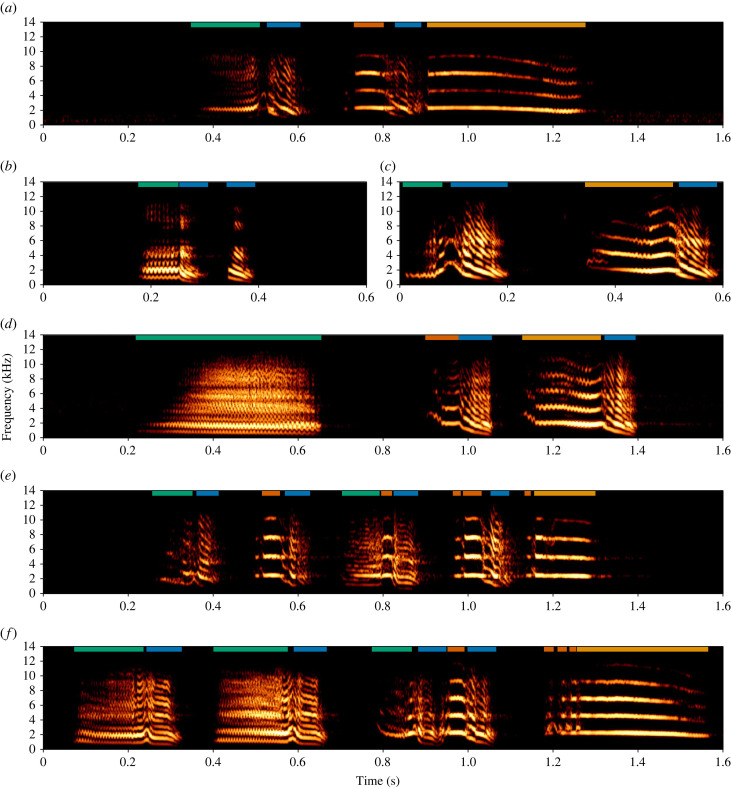
(*a*–*f*) Examples of magpie combinatorial structures, ranging in number from two (*a*–*c*) to five (*e*) distinct comprising calls. Calls within combinations are separated by silent periods, and segment boundaries are defined by smaller silent periods or sudden spectral shifts. Coloured bars delineate boundaries of the segments comprising calls, where colour denotes classes of acoustically similar segments: ‘down sweep’ (DS) (blue), ‘long high’ (LH) (yellow), ‘noisy line’ (NL) (green) and ‘short high’ (SH) (orange).

Classification of combinations and comprising vocal units was based on a method previously used for this species (see [39,40]) as follows: discrete (non-combined) calls are isolated vocal units separated by silent periods over 0.5 s, while combinations refer to vocal sequences containing calls separated by silent periods equalling or less than 0.5 s (figure 1). To verify this method, we allowed inclusion of non-song combinations containing calls separated by silent period(s) slightly deviating over 0.5 s (up to 0.4 s). We found that much larger silent periods separated discrete calls and combinations (greater than 1 s) and that calls comprising combinations largely fell into the previously suggested arrangement, with a maximum period for between-call silence of 0.612 s and mean of 0.113 s (±0.09 s). In pilot investigations, we found calls appear to comprise smaller vocal units (segments) that are combined in varying arrangements to produce seemingly acoustically distinct calls (figure 1). Segments, thus, were defined according to separation by small silent periods [49], or by sudden spectral shifts [50] if observed as an isolated segment (surrounded by silence) in any call. For this study, the maximum period of between-segment silence was 0.025 s and the mean was 0.008 s (±0.006 s). It should be clarified that it was not taken into account whether segments or calls occur as isolated vocal units outside combinations. Although some are known to commonly occur as isolated units [39,40,42], here, the study's aim was to investigate and describe the acoustic properties and transitions occurring within the magpie combinatorial repertoire.

### 2.4. Praat annotation

Recordings were annotated to delineate start and end time boundaries of distinct combinations and their respective comprising vocal units using Praat 6.1.16 [51]. Time boundaries for (i) combinations, (ii) calls and (iii) segments were annotated on distinct tiers, respective to combinatorial level. In cases where only one segment comprised a call level unit, boundaries were defined for the single unit on both the segment and call tier. In cases where the boundaries of two segments extend over one another at the point of transition, boundaries for each segment were placed at the point at which the intersection ceased for each respective segment (see electronic supplementary material, figure S1 for an example).

Segments were assigned labels according to spectrographic appearance, with original descriptions being: ‘down sweep’ (DS), ‘high line’ (HL), ‘long high’ (LH), ‘noisy line’ (NL), ‘short high’ (SH) and ‘up sweep’ (US). Following evaluation of clusters for the initial UMAP analyses on all segments, some segment hand label classes which consisted of a low number of samples (*N* < 50 samples) were combined into other hand label classes due to an observed overlap of clusters (electronic supplementary material, figure S2). The overlap of two segment classes across a cluster suggests little acoustic difference between the two classes. As such, for those segment classes which overlapped with another larger class, the label for the class with fewer samples was modified to the label of the larger segment class to which it appeared to instead belong (namely HL to LH and US to SH). Accordingly, final segment labels comprised: ‘down sweep’ (DS), ‘long high’ (LH), ‘noisy line’ (NL) and ‘short high’ (SH). Segment labels were then used to categorize calls and subsequently combinations (i.e. ‘NL-DS LH-DS’ describes a combination wherein the first call contains a NL segment preceding a DS segment, and the second call comprises an LH segment followed by a DS segment; figure 1). Annotations, including labelled vocal units with start and end time boundaries, were exported in text-grid format, along with their respective recording (44.1 kHz sampling rate; 16-bit sampling depth), for later creation of segment and call datasets.

### 2.5. Final sample size

In total, the recordings used in this study contained 1644 segments comprising 697 calls and 273 combinations. Two individuals had much higher counts of combinations compared with any other individual in the dataset. As such, a smaller portion of combinations from each of these two individuals was randomly selected for analysis, to reduce the risk of bias due to over-representation (electronic supplementary material, table S1). Random sampling was also used prior to the SH segment within-class analysis (see below) to reduce spectrograms for two individuals with much higher counts compared with any other individual (electronic supplementary material, table S3). Consequently, the final sample size for analysis included 1333 segments comprising 561 calls and 222 call combinations from 23 individuals in 16 groups. Additionally, these combinations were comprised at least two and up to five calls, and calls contained segments which ranged in number from one to seven.

### 2.6. Data preparation

Data preparation and analysis were undertaken in Python v. 3.6.5 and largely followed the method outlined in [52], with some modifications to account for acoustic parameters specific to magpies [53]. All recordings were filtered using a Butterworth band-pass filter set at 0.4–8 kHz, to encompass the frequency range in which most of the spectral content lay, when considering all vocalizations. Spectrograms were created using the absolute value taken from a one-sided short-time Fourier transformation. A Mel filterbank was used to logarithmically scale the spectrograms, set at the same frequency range as used in the Butterworth band-pass filter. Datasets were created for (i) segments and (ii) calls by locating the respective vocal units within spectrograms, being automatically assigned according to the defined time boundaries in the exported text-grid annotation files. Each segment or call spectrogram was padded to the same length (UMAP requires a fixed number of attributes) by log-rescaling in time (relative to the log-duration of the segment) and zero-padding to a uniform size (64 frequency and 64 time components) [52,54].

### 2.7. Dimension reduction projections

We used the dimension reduction method UMAP to project cut-spectrograms into latent space where each time-frequency bin was treated as independent dimensions, using the Python package *umap-learn* [55]. UMAP creates a nearest neighbour graph from the spectrograms by computing the distance between time-frequency bins, and then finding a low-dimensional representation while preserving the structure of the graph (through an iterative optimization procedure). With the exception of minimum distance (recommended to be set to zero for clustering), we found the default UMAP parameters adequate for our dataset (also suggested in [52,54]). In this paper, we show projections for spectrograms of (i) all segments, (ii) each segment class and (iii) all calls (but see electronic supplementary material) [53].

### 2.8. Evaluation of clusters

We computed the silhouette score (*S*) to measure the extent to which the distribution of spectrograms (within and between clusters) could be described by class membership for given predictor variables [56] (*sensu* [52,54]), namely: hand label (see above for categories), caller identity, group identity, study site and sex. To calculate *S* for a predictor, the silhouette coefficient for each spectrogram was measured. The silhouette coefficient indicates a spectrogram's closeness to other data within the same predictor class, relative to the distance to the nearest data point in another class. *S* was then calculated as the mean silhouette coefficient across the whole projection on a scale from −1 to 1 [52,54,56]. A score of 1 indicates the predictor describes the model perfectly (all class members grouped into their own cluster and each cluster is well separated), a score of 0 indicates overlapping clusters and a score of −1 indicates inadequate description of data distribution [56]. We used the Kruskal–Wallis H-test (H) to compare distribution of a predictor with a distribution expected by chance, by comparing *S* with that generated for randomly permuted labels (*sensu* [52,54]). Thereby, we determined whether the distribution of data for any given predictor is significantly different from what was seen in a randomly generated projection [57].

For the within-segment class analysis, we also analysed whether spectrogram distribution across clusters could be explained by the segments' position within a call, relative to its arrangement with neighbouring segments. Categories were created separately for each segment class based on common associations with neighbouring segments comprising the same calls. For example, for the LH segment class, we grouped data into the following two categories: (i) segments preceding a DS segment within a call and (ii) segments positioned at the end of a call or that solely comprise a call unit. Thereby allowing determination of whether variation in segment position correlated with a quantitative change in its acoustic structure.

For projections on calls, we analysed whether spectrograms clustered according to the composition of their comprising segments. To do so, we created groupings of calls based on any associations found between the segments comprising calls within each cluster. As such, we created three groups: (i) calls containing an LH segment, (ii) lone NL segment calls, and (iii) all other calls (namely, lone DS/SH segments, or any variation of these two segments and/or the NL segment in combination).

To investigate whether there was further separation of call types within each of the three call groups (see above), we conducted the following separate UMAP analyses on (i) calls containing an LH segment, (ii) calls comprising any combination of, or any lone, DS, NL and SH segments, and (iii) calls comprising DS or SH lone segments, or any combination of DS, NL and/or SH. Because many calls contained segments that were repeated, we used a simplified call labelling system which excluded display of repeated consecutive segments. For example, a call comprising three SH segments followed by a LH segment would be given the label SH-LH. Per projection, we created labels for groupings of similar call types, based on associations found in the distribution of spectrograms in the model (see electronic supplementary material).

### 2.9. Cluster analysis limitations

It was not possible to project a sufficiently large dataset in which the number of spectrograms for segments and calls was balanced between individuals. As such, to establish whether repeated measures created a bias in results, we used silhouette and Kruskal–Wallis H-test scores to measure if individual identity was a significant predictor for variation between spectrograms. If spectrograms were not observed to cluster by individual identity (indicated by silhouette score) and if the distribution of data from all individuals is not significantly different to what would be observed in a randomly generated projection (indicated by Kruskal–Wallis H-test), this suggests vocalizations did not differ strongly between individuals. Thereby, data was not clustering by individual identity and thus feasibility of including repeated measures in the analysis was supported. We also ensured no one individual had significantly more spectrograms included in each projection in comparison with other individuals, to avoid bias due to over-representation (electronic supplementary material).

### 2.10. Sequential transition

We assessed first-order transition probabilities to attain a statistical measure of directionality in segment and call transition. In this way, we could determine whether transition between segments and calls occurred in a predictable and repeatable way across all individuals and groups which provides insight into whether there are trends for changes in sound patterning at both combinatorial levels. As such, we investigated transitions between (i) segments in forming calls and (ii) calls in forming combinations. To do this, we extracted forward transition probabilities to create a probability matrix between classes of (i) segments and (ii) calls, whereby the probability of transition is dependent upon the preceding vocal unit [52,58].

We conducted randomized simulations (*sensu* Monte Carlo simulations; for examples in other vocal studies, see [59,60]) to determine whether the observed transition probabilities were significantly different to that expected by chance across (i) calls and (ii) combinations. For each of the 10 000 iterations, we randomized the assignment of (i) segments to calls and (ii) calls to combinations, such that the comprising vocal unit's frequency of occurrence reflected that in the original dataset. For each iteration of randomly generated calls/combinations, we calculated the transition probabilities between vocal units, thereby generating null distributions against which the significance of the observed probability values was compared. Significance was calculated as the relative ranking of each observed transition probability among the sample values for the same statistic in the randomized iterations. The observed probability was found to be significantly different from chance if it occurred outside the 95th percentile of values in the randomized data (two-tailed).

It should be noted that the analysis used here investigates first-order transitions and does not account for longer range dynamics [52,60] in magpie combinatorial structures. For the purposes of this study, we found consideration of first-order transitions to be a suitable means of describing sequential patterns to investigate the potential for organization in the combining of magpie segments and calls. Sequential transition analysis was performed in Python 3.8.13 [53], using a modified version of the code outlined in [52] and the package *NetworkX* [61].

## 3. Results

### 3.1. Segment analysis

**Figure 2.**
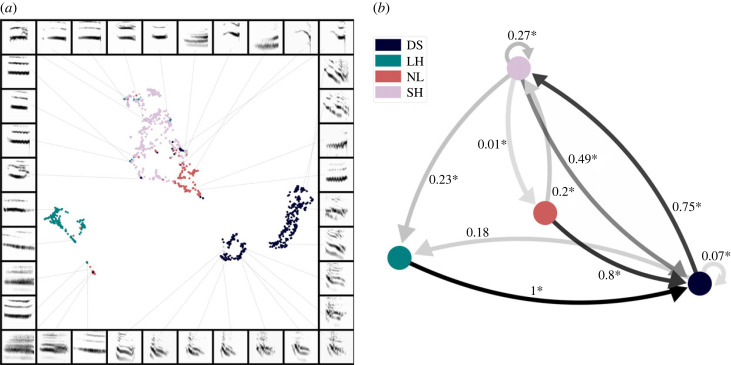
(*a*) Segment spectrograms projected into UMAP latent space (*N* = 1333). The four classes of hand labels separate into largely distinct clusters which were supported by the silhouette score (*S* = 0.481); this was well above chance. The legend in (*b*) can be applied to similarly coloured data in (*a*). Segment colour codes: DS (dark blue), LH (teal), NL (red) and SH (light purple). (*b*) Transitions between segment classes (coloured circles). Arrowed lines represent transitions between segments with opacity increasing in relation to transition probability (value indicated by text next to respective lines; asterisk indicates probability was significantly different to that expected by chance).

Spectrograms of segments (smallest combinatorial unit; *N* = 1333) clustered well in latent space according to the given hand labels (DS, LH, NL and SH; see Methods). Silhouette score indicated hand labels most accurately described clusters (*S* = 0.481) and was significantly different from a randomly generated distribution (H(2) = 1397.166; *p* < 0.001; figure 2*a*). Distribution was significantly different from random for both sex (H(2) = 78.110; *p* < 0.001) and study site (H(2) = 82.543; *p* < 0.001); however, the difference was not major and silhouette score suggested these factors minimally described distribution across clusters (sex: *S* = −0.001; study site: *S* = 0.029). All other predictors did not correlate with the distribution of spectrograms across clusters (individual identity: *S* = −0.202; group identity: *S* = −0.171) and were not significantly different to random (individual identity: H(2) = 0.021, *p* = 0.884; group identity: H(2) = 0.379, *p* = 0.583). Because individual identity was an inadequate predictor of the data distribution across clusters, we did not find reason against pooling spectrograms from all individuals into the same projection.

Due to the difference in the number of spectrograms per assigned segment class, another UMAP analysis was run on a balanced dataset (*N* = 173 spectrograms per class; based on count in the smallest segment class; see electronic supplementary material) to ensure there was no bias in the UMAP results (electronic supplementary material, figure S3). The balanced analysis revealed slightly more separation between clusters of segment classes, reflected in a marginally higher silhouette score (*S* = 0.505) which was also significantly above chance (H(2) = 772.588; *p* < 0.001). In comparing the UMAP analyses on the balanced and unbalanced dataset, there was minimal difference between the silhouette scores for hand labelling, thus suggesting that distinction between segments can still be reliably assessed in the unbalanced dataset. Therefore, results support the hand labelling classifications and show that segment spectrograms separate into four distinct classes.

We found evidence for organization in transition between segments, which appear to be predictably arranged to form calls (figure 2*b*). The LH segment class displayed the most extreme directionality, with its only observed transition being to DS (probability of 1; *p* = 0.0001). Similarly, NL typically transitioned to DS (0.8 probability; *p* = 0.0001), occasionally to SH (0.2 probability; *p* = 0.0002), but never to LH or NL (*p* = 0.0001). DS predominantly transitioned to SH (0.75 probability; *p* = 0.0001) and less frequently to LH (0.18 probability; the only segment transition that was not significantly different to that expected by chance: *p* = 0.09). Furthermore, DS displayed transition directionality, rarely transitioning to DS (0.07 probability; *p* = 0.0001) and never to NL (*p* = 0.0001). Lastly, SH most frequently transitioned to DS (0.49 probability, *p* = 0.0001), less to either SH (0.27 probability; *p* = 0.0002) or LH (0.23 probability; *p* = 0.0001), and very rarely to NL (0.01 probability; *p* = 0.0001). Thus, SH displayed the least directionality, being observed to transition to every segment class; however, transition probability varies between classes and each observed probability was significantly different to random. Overall, these results provide evidence for predictable organization in the combination of segments to form calls.

To determine whether these findings are reflected at the individual level, we separately analysed the combinatorial repertoire for two individuals (both female; see electronic supplementary material). Each individual's findings reflected that shown when pooling all individuals, for both segment class distinction (electronic supplementary material, figures S4A and S5A) and sequential transition (see electronic supplementary material, figures S4B and 5B). Furthermore, there were no cases of misclassification of segments when conducting UMAP on a single individual's repertoire. This may suggest the cases of misclassification observed in figure 2*a* may be a side-effect of pooling all individuals onto the same projection. Critically, these findings show the distinction between classes of segments and the trends in segment transition that are observed when pooling all individuals are also reflected at the individual level.

### 3.2. Within-segment class analysis

**Figure 3.**
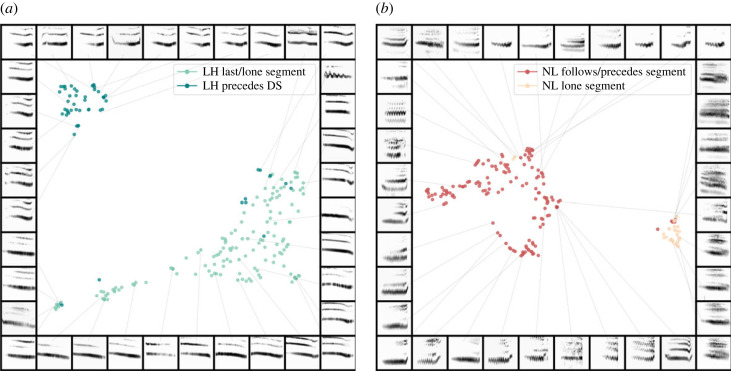
Spectrograms from two separate segment classes projected into UMAP latent space. (*a*) UMAP on LH segments (*N* = 203) showing clear separation into two clusters. Cluster separation correlates with variation in arrangement of LH compared with neighbouring segments. Namely, LH segments positioned at call end, or which solely comprise a call (light green), versus LH segments preceding a DS segment (teal; *S* = 0.49). (*b*) UMAP on NL segments (*N* = 173), showing clear clustering according to the whether the segment is produced in isolation (separated by silence; red) or in combination with other segments within a call (light orange; *S* = 0.581).

UMAP analyses on spectrograms for each segment class revealed further separation within some segment classifications (figure 3; electronic supplementary material). For LH (*N* = 203), location of the study site slightly correlates with data distribution across the two apparent clusters (*S* = 0.096) and was significantly, albeit slightly, different from chance: H(2) = 23.022; *p* < 0.001 (electronic supplementary material, figure S6A). This suggests there may be spectrographic acoustic differences between individuals in the Crawley site versus individuals in Guildford. However, the position of LH relative to neighbouring segments within the call (see Methods) best describes the distinction between the two main clusters in the LH projection (*S* = 0.490, H(2) = 230.923; *p* < 0.001; figure 3*a*). One cluster entirely comprised LH segments that precede a DS segment (within their respective call), with all bar two of the spectrograms being produced by groups located in Guildford. The other cluster predominantly comprised all LH segments positioned at the end of the call or which solely comprise the call. These results suggest there is an acoustic change to the LH segment when it precedes DS, resulting in separation of spectrograms. Furthermore, LH-DS containing calls appear to be produced by Guildford groups (*N* = 56) more frequently than Crawley groups (*N* = 5), which probably explains the correlation between study site with the clusters in the model. Thus, rather than study site, the position of the LH segment (namely whether it directly precedes a DS segment) best describes the distinction between clusters of spectrograms (figure 3*a*). No other predictor described distribution of spectrograms across clusters (*S* < 0).

Similarly, for NL segments (*N* = 173; figure 3*b*), position (i.e. relative to arrangement of neighbouring segments) described most of the separation between the two clusters (*S* = 0.581) and was well above chance (H(2) = 218.568; *p* < 0.001). One cluster was largely composed of lone NL segments (the segment itself making up a call level unit), whereas the other cluster predominantly contained NL segments in combination with other segments in a call (figure 3*b*). Therefore, it appears NL segments are acoustically modified by the presence of neighbouring segments, which largely explains the distinction between spectrogram clusters within this segment class. Modification appears to occur regardless of whether NL is preceding or following the segment(s) with which it is combined. No other predictors better-described spectrogram distribution in the model (*S* < 0.028; electronic supplementary material, figure S6B). The within-segment class variation for LH and NL segments was similarly observed in the individual combinatorial repertoire analysis (see electronic supplementary material, figure S7).

Defining within-segment class variation was not as clear-cut for the two other segment classes. SH segments (*N* = 427) slightly clustered by study site (*S* = 0.093); significantly better than chance (H(2) = 104.702; *p* < 0.001). However, clusters for each site were not well defined or separated (electronic supplementary material, figure S6C), and no other predictor explained distinction between SH spectrogram clusters (*S* < 0). Likewise, DS segments (*N* = 468) separated into two clusters (electronic supplementary material, figure S6D); however, the separation cannot be described by any of the predictors (*S* < 0.036).

### 3.3. Call analysis

**Figure 4.**
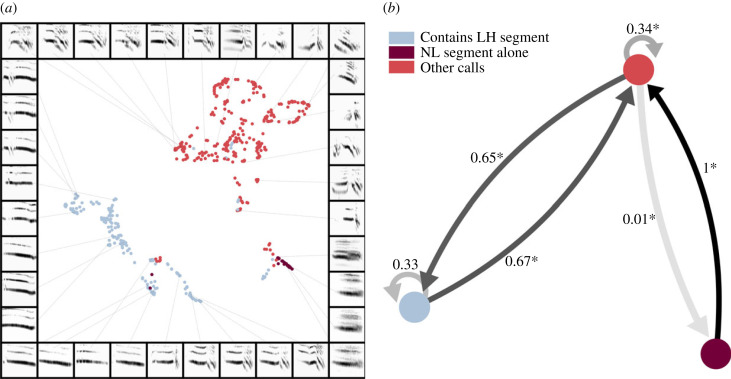
(*a*) Call spectrograms (*N* = 561) projected into UMAP latent space, forming three largely distinct clusters which correlate with the comprising segment composition (*S* = 0.445); significantly different from chance. The legend in (*b*) can be applied to the similarly coloured data in (*a*): (i) calls containing lone DS/ SH segments, or these segments and/or NL in any combination (red; e.g. SH-NL-DS), (ii) calls containing LH (dark purple; e.g. SH-DS-LH) and (iii) lone NL calls (i.e. the segment itself comprising a call level unit; blue). (*b*) Transitions between the groups of calls observed in (*a*) (symbolized by coloured circles). Arrowed lines represent transitions between calls with opacity increasing in relation to transition probability (value indicated by text next to respective lines; asterisk indicates probability was significantly different to that expected by chance).

Call spectrograms (*N* = 561) largely were separated into three clusters (figure 4*a*), which seem to coincide with findings of within-segment class variation for the NL segment. Furthermore, distinction between clusters appeared to correlate with the segment transition analysis results, as distinction between clusters was largely due to variation between calls based on comprising segments. Accordingly, call spectrogram clusters can largely be described as three broad groupings: (i) calls comprising lone DS or SH, or any combination of SH, DS and/or NL, (ii) calls containing LH, and (iii) lone NL calls. The silhouette score for this way of grouping calls was moderately high (*S* = 0.445) and was significantly different from random (H(2) = 552.825; *p* < 0.001).

A significant, albeit marginal, difference was observed for study site (H(2) = 23.423; *p* < 0.001), suggesting the acoustic structure of calls may be distinct between study sites, although this was not reflected in the silhouette score (*S* = 0.027). Silhouette score for all other predictors did not adequately describe call spectrogram distribution across clusters: individual identity (*S* = −0.278), group identity (*S* = −0.199) and sex (*S* = 0.010). Furthermore, silhouette scores did not significantly differ from random for individual identity (H(2) = 3.127; *p* = 0.077), group identity (H(2) = 0.105; *p* = 0.746) and sex (H(2) = 2.283; *p* = 0.131).

There is evidence for predictable organization in call combining, and most transition probabilities between call groups were significantly different from random (figure 4*b*). Transition analysis revealed that calls comprising lone DS or SH, or any combination of DS, NL and/or SH, mostly preceded calls containing an LH segment (0.65 probability; *p* = 0.0001). Less frequently, transition occurred to calls within the same group (0.34 probability, *p* = 0.0001) and very rarely to lone NL calls (0.01 probability, *p* = 0.0001; figure 4*b*). Lone NL calls only ever transitioned to calls composed of either lone DS or SH segments, or any combination of DS, NL and/or SH segments (probability of 1; *p* = 0.007). Accordingly, lone NL segment calls never transitioned to calls within the same group, however, this was not significantly different to chance (*p* = 0.485). Finally, calls containing LH never transitioned to lone NL segment calls (*p* = 0.002) but were most likely to transition to calls comprising either lone DS or SH, or any combination of DS, NL and/or SH (0.67 probability; *p* = 0.022). Results therefore suggest predictable call transition within the production of non-song call combinations. Overall, only two call transition probabilities were not significantly different to probabilities generated in random simulations.

### 3.4. Within-call group analysis

Using UMAP, we conducted separate projections for spectrograms within the three broad call groups (see above) to determine whether there was any within-group separation of calls. Overall, results suggest some clustering of acoustically distinct call types, as well as some differences in the distribution of spectrograms between study sites; however, in each case, clusters are not well defined (see electronic supplementary material, figures S8 and S9). Thus, it is likely that the current sample size is not large enough to reveal such complexities with strong statistical certainty.

## 4. Discussion

In this study, we show that the magpie repertoire can be broken down into four distinct segment classes that are produced across all groups and at both of the study site locations. These segments occur together in predictable ways to create calls and are the building blocks of observed vocal combinations. Clusters of similar spectrograms correlated most with the assigned hand labels for segments compared with any other measured variable and suggest our method of hand labelling is accurate in classifying magpie vocalizations.

There appears to be further clustering within two segment classes, and analyses suggest distinction here correlates with variation in the positioning of the segment within the call, in relation to presence and arrangement of neighbouring segments. Rather than indicating a need for further classification of segment classes (i.e. assigning distinct class labels to each cluster), instead this may reflect variation due to coarticulation. In human speech, coarticulation is categorized as alterations in an acoustic signal which is often caused by the positioning or overlap of neighbouring phonemes [62]. For example, the effect of coarticulation can be observed in the subtly different forms the phoneme /s/ holds in the words ‘***s****ail’* and ‘*boot**s**’*, which is largely a consequence of variation in the required positioning of articulatory structures [62]. Similarly in this study, results suggest that modification of a segment can occur depending on its arrangement in relation to neighbouring segments within a call and resulting in within-segment class differences. Currently, there is little research on coarticulation in animal systems (but see [63–65]); however, these findings suggest the potential for this effect should be considered when undertaking acoustic analyses to classify animal vocal units.

Critically, results show structure at both the segment and call level, suggesting magpies are capable of combinatoriality at two levels. Segments appear to be predictably combined to form discrete calls which are then arranged to form longer vocal structures, and all excluding one of these transition probabilities are significantly different to what is expected by chance. Transition analysis indicates that there is variation in the probability for segments to transition to other segments, depending on the immediately preceding segment. For example, the SH segment may transition to any other segment (albeit very rarely to NL) and may be transitioned to by either a DS, SH or NL preceding segment. On the other hand, the LH segment only ever transitions to a DS segment and will follow only the DS or SH segments. These results suggest the combining of segments follows some ordering rules and, therefore, segment arrangement probably has a functional importance in the production of magpie calls. Furthermore, combining of calls from the three broad call classes occur in a predictable and repeatable way, and this is present across all study groups.

Structured arrangement has been demonstrated in non-song systems for other species at either the within- or between-call level [25,27,28,30,66,67] (for reviews see [5,7,31,68]). Existing evidence suggests non-human species are restricted to combining at only one of these levels [4,32,34]. Our findings are distinct in that they show magpies can combine distinct types of vocal units on more than one level, and the precise arrangement appears to be governed by ordering rules. To our knowledge, to date there is only one other suggestion for multi-level complex combinatoriality in a non-human species, that being hierarchically structured vocal call combining in chimpanzees [35]. Chimpanzees have shown a capacity to combine calls (which are independently produced) to form two-unit sequences (bigrams), which are then predictably recombined to produce three-unit sequences (trigrams; [35]). Of note, the study included quantitative analysis on the species' full combinatorial repertoire as was similarly undertaken for our analysis here, opening the door for future work to incorporate whole combinatorial repertoires (rather than focusing on a small selection). Together with our findings, this demonstrates that a capacity for multi-levelled complex combinatoriality (i.e. combining across different combinatorial levels in magpies, or hierarchical combining within the same combinatorial level in chimpanzees), is present outside human language. More specifically, such comparative findings can help inform understanding regarding the various communicative mechanisms employed by non-human animals, in this case combinatoriality, in order to generate distinct (and perceptually salient) signals.

Interestingly, neither individual nor group identity described distinction between clusters in any of the analyses. Furthermore, the distribution of spectrograms from the same individual or group was not significantly different from a random distribution, relative to the distribution of data from other individuals or groups in the model. As classes of segments and calls do not appear to be largely distinct between individuals and groups, and as segment and call arrangement appears to follow some ordering rules, any attached meaning is probably generalized for the subspecies [69]. Consequently, there is suggestion that classes or combinations of segments and calls may possess function(s) relating to contexts that: (i) all individuals may encounter, (ii) which require transmission of specific familiar information, and (iii) for which it is advantageous to the caller to have conspecifics respond in a predictable manner, as well as being advantageous for the listener to clearly perceive the message being relayed (for example: an alert call, a signal to mob a predatory threat or a signal to recruit group members to the caller). Such a result is of note, as it provides support for our hypothesis that order and composition of segments and calls within combinatorial structures carry a functional importance for magpies. Validation through playback experiments is required, however, prior to any inference as to whether variation in segment and call arrangement encodes qualitative change in meaning at the call and combination levels, or, respectively, to determine the factors that result in the combinatorial complexity governing the magpie combinatorial repertoire. Furthermore, comparison with magpie populations situated in other areas (within the Western Australian magpie range) would shed further light on how stereotyped the combinatorial dynamics of Western magpies are or whether there exists any more subtle group-specific variation.

In this study, we have shown that magpie calls comprise smaller sound segments and there appears to be a level of predictability in arrangement. Furthermore, the resultant calls are productively combined to form longer vocal structures. Current research suggests animals are restricted to the combining of up to two or three meaningful vocal units [19,34,35]; however, in initial investigations, we found that magpie non-song call combinations can be composed of up to five calls [42]. Exploration into the meaning of segments and calls, as well as investigation into transitional probabilities encompassing more than two states, will allow understanding on the extent of combinatorial complexity present and what function it holds in this system [6,27,49]. This study provides significant insight into the magpie communication system and suggests magpies display combinatoriality on two levels (at both the within- and between-call level), for which there is currently little to no evidence outside of human language [4,32,34]. Such a novel finding contributes to the ever-expanding array of comparative research that highlights combinatorial capabilities of non-human species which, in turn, may help to shed light on the evolution of combinatoriality within human language.

## Data Availability

Data, including audio (WAV) and annotation (Praat textgrid) files, can be accessed at the research repository for the University of Western Australia: https://doi.org/10.26182/s77t-hw04. Code for data analysis and production of figures was sourced from Sainburg *et al*. ([52]; https://github.com/timsainb/avgn_paper). Code specific to the data analysis and figures found in this paper can be accessed at https://github.com/sarahLwalsh/avgn_paper (figure and result output not displayed as per Royal Society Interface Author Guidelines).
